# Predatory mites *Amblydromalus limonicus* and *Amblyseius herbicolus* (Acari: Phytoseiidae) as potential biocontrol agents of *Eotetranychus sexmaculatus* (Acari: Tetranychidae) in avocado: examining predation on different prey life stages

**DOI:** 10.1093/jee/toaf036

**Published:** 2025-03-29

**Authors:** Yuhao Yang, Keshi Zhang, Zhi-Qiang Zhang

**Affiliations:** School of Biological Sciences, University of Auckland, Auckland, New Zealand; Manaaki Whenua – Landcare Research, Auckland, New Zealand; School of Biological Sciences, University of Auckland, Auckland, New Zealand; Manaaki Whenua – Landcare Research, Auckland, New Zealand; School of Biological Sciences, University of Auckland, Auckland, New Zealand; Manaaki Whenua – Landcare Research, Auckland, New Zealand

**Keywords:** biological control, Manly’s preference index, non-invasive, interspecies interaction, six-spotted spider mite

## Abstract

The six-spotted spider mite, *Eotetranychus sexmaculatus* (Riley) (Acari: Tetranychidae), is a severe pest of avocado trees, causing excessive leaf drop and reduced yield. Two predators found to be sympatric with *E. sexmaculatus* in surveys, *Amblydromalus limonicus* (Garman & McGregor) and *Amblyseius herbicolus* (Chant) (Acari: Phytoseiidae), may serve as potential biological control agents. We examined their potential in 2 laboratory experiments. (i) In no-choice tests, each predator was presented with 40 eggs, 40 larvae, 40 nymphs, or 20 female adults of *E. sexmaculatus*, and predation and oviposition rates (per day) were measured. (ii) In choice tests, predators were given a mixture of 10 eggs, 10 larvae, 10 nymphs, and 10 female adults, and the same parameters were measured, but with prey stage preference also assessed using Manly’s preference index. Our results showed both *A. limonicus* and *A. herbicolus* fed and reproduced on all stages of *E. sexmaculatus*. Significantly higher predation rates were observed for *A. limonicus* on *E. sexmaculatus* than *A. herbicolus*. Both predator species preferred earlier life stages of *E. sexmaculatus*, with the preference more pronounced in *A. herbicolus* than in *A. limonicus*. In both choice and no-choice experiments, the oviposition rate (one criterion for determining whether artificially released biological control agents can use the nutrients of the new prey to reproduce) was higher in *A. limonicus* than *A. herbicolus*. This study is the first to report on the biological control potential of these two predatory mite species for use against *E. sexmaculatus* in avocado plantations.

## Introduction

Avocado (*Persea americana* Mill.) is a unique tropical and subtropical plant from Central America, with fruit that has gained popularity worldwide as a nutrient dense and healthy food source (Ayala Silva and Ledesma 2014, Talavera et al. 2023). The unique chemicals within avocado fruit and their derivatives are used in the manufacture of some industrial products, such as guacamole (Majid et al. 2020, García et al. 2021, Talavera et al. 2023). The direct and indirect damage to avocado quality caused by pests feeding on its fruit and leaves has caused serious economic losses to the avocado industry worldwide (Erichsen and Schoeman 1992, Hoddle et al. 2003, Ramírez-Gil et al. 2019, Torres et al. 2023). One phytophagous pest that feed on avocado leaves, the six spotted spider mite *Eotetranychus sexmaculatus* (Riley) (Acari: Tetranychidae), has had a serious negative impact on the avocado growing industry (Hoddle and Morse 2012, Bragard et al. 2023). Infestation of avocado trees by *E. sexmaculatus* usually occurs in late spring and early summer, and occasionally in autumn (Froud et al. 2002, Bragard et al. 2023). The pests are usually found on the lower surface of leaves, colonizing along the midrib and main veins of the leaf (McMurtry 1989, Lu et al. 2016). After infesting an avocado leaf, *E. sexmaculatus* feed on the phloem with their piercing sucking mouthparts, causing yellowing of the leaves and purple spots at the feeding sites. Feeding damage caused by *E. sexmaculatus* can lead to severe defoliation and a subsequent decline in avocado yield (Stevens et al. 2001, Jamieson and Stevens 2007).

Currently, the most used method of combating *E. sexmaculatus* damage to avocado plantations is chemical control with broad-spectrum pesticides, such as abamectin, milbemectin, or fenpyroximate (Froud et al. 2002, Steven 2004). Although chemical control works rapidly to reduce population densities at a low cost, the toxicity of broad-spectrum pesticides to natural enemies of *E. sexmaculatus* may lead to more serious management issues, such as the recolonization of *E. sexmaculatus* in the future, and the resistance of *E. sexmaculatus* to pesticides should also be considered (Tomkins 2002, Learmonth 2019). A recent report on the number of *E. sexmaculatus* on avocado leaves that were either sprayed or unsprayed with pesticides, confirms and reinforces these concerns about side effects on natural enemies and pesticide resistance (Logan et al. 2023). Although some studies have targeted plant resistance (including its molecular mechanisms) as another method for *E. sexmaculatus* control (Lu et al. 2016, Liang et al. 2020, Wang et al. 2023), these approaches have not been proven on avocados. Therefore, other available control methods are urgently needed.

Compared to other control strategies that have been utilized for spider mites, biological control is considered an environmentally friendly alternative (McMurtry et al. 2015, Knapp et al. 2018, Assouguem et al. 2022). Predatory mites in the family Phytoseiidae (Acari: Mesostigmata) are important biological control agents of many spider mite pest species on a variety of crops (McMurtry et al. 2013, Tixier 2018, Wang et al. 2024). Previous studies have confirmed that some phytoseiids are effective against *E. sexmaculatus*. For example, Hao et al. (2021), and Chen et al. (2023) found that *Neoseiulus barkeri* Hughes and *Amblyseius swirskii* Athias-Henriot were effective predators of *E. sexmaculatus* in rubber trees and were good candidate biological control agents for field releases. Caceres and Childers (1991) showed that *Galendromus helveolus* (Chant) could develop to adults with *E. sexmaculatus* as the only food source on citrus crops in Florida. However, because they have not been commonly found in wild avocado leaves until now, their effectiveness and adaptablity in artificial release is in doubt (McMurtry et al. 2013, Learmonth 2019, Logan et al. 2023), and its potential threat for biological invasion is also worth considering (Babendreier 2007, Hajek et al. 2016).

In a recent survey of *E. sexmaculatus* and its potential predators on sprayed and unsprayed “Hass” avocado trees in New Zealand, 2 predatory mite species were commonly sympatric with *E. sexmaculatus*: *Amblydromalus limonicus* (Garman & McGregor) (Acari: Phytoseiidae) and *Amblyseius herbicolus* (Chant) (Acari: Phytoseiidae) (Logan et al. 2023). *Amblydromalus limonicus* is a type III-b generalist predator that can feed and reproduce on a wide range of prey (McMurtry et al. 2013) and was reported as a potential predator of the phytophagous mite, *Oligonychus punicae* (Hirst) (Acari: Tetranychidae), as early as the 1960s (McMurtry and Scriven 1971). Compared to other predatory mites of the family Phytoseiidae, the commercialization of *A. limonicus* in the Americas, Europe and Oceania regions (Knapp et al. 2013, Schoeller et al. 2020), as well as its natural association with avocado leaves, makes it a good potential biocontrol agent against *E. sexmaculatus* on avocado. Previous studies have shown *A. limonicus* to be an effective biological control agents for thrips (Knapp et al. 2013, Lam et al. 2019, Schoeller et al. 2020, Mouratidis et al. 2023), psyllids (Liu et al. 2019), and whiteflies (Knapp et al. 2013, Cuthbertson 2014, Medd and GreatRex 2014, Lee and Zhang 2018), as well as some spider mite species (Knapp et al. 2013, Samaras et al. 2019, Wang et al. 2024). Its potential role as a biological control agent against *E. sexmaculatus* is unknown and worth investigating. *Amblyseius herbicolus* is also a type III-c generalist predator (McMurtry et al. 2013), and it is the most common predatory sympatric species for *E. sexmaculatus* in avocado orchards in New Zealand (Logan et al. 2023). In recent years, *A. herbicolus* has been shown to be a potential biological control agent for some pests, such as mites (Reis et al. 2007, Rodríguez-Cruz et al. 2013), whiteflies (Xin and Zhang 2021, Cardoso et al. 2024), psyllids (Jorge et al. 2021), and thrips (Lam et al. 2019). Fortunately, Zhang and Zhang (2021) provided a better method for large-scale cultivation of *A. herbicolus* by using *Carpoglyphus lactis* (Linnaeus) (Acari: Carpoglyphidae) as food, which is conducive for the potential large-scale commercialization of *A. herbicolus.*

Additionally, both *A. limonicus* and *A. herbicolus* can develop and reproduce on supplementary food, such as pollen, which makes it possible for predators to remain on avocado leaves when *E. sexmaculatus* population densities are low (Liu and Zhang 2017, Xin and Zhang 2021).

Although *A. limonicus* and *A. herbicolus* have been found to inhabit avocado leaves when *E. sexmaculatus* are present, it is unknown whether they can attack all stages of this prey. Therefore, this study simulates the artificial release of predators in a controlled laboratory environment with modified Munger cells and addresses the following objectives: (i) In no-choice tests, determine whether *A. limonicus* or *A. herbicolus* can prey on the single life stages of *E. sexmaculatus* and determine the rate of predation over 24 h. (ii) In choice tests, determine the predation preferences of *A. limonicus* or *A. herbicolus* for *E. sexmaculatus* at different life stages and the rate of predation over 24 h in an arena with mixed life stage of *E. sexmaculatus*. (iii) In both choice and no-choice tests, determine the number of eggs produced by *A. limonicus* or *A. herbicolus*, to assess whether these species can reproduce and thus demonstrate their potential as sustainable and environmentally friendly biological control agents.

## Materials and Methods

### Mite Cultures

#### Prey Mites

The dry fruit mite *C. lactis* was used for rearing predatory mites, and originated from a commercial supplier (Bioforce Limited, Karaka, Auckland, New Zealand) (Zhang and Zhang 2021). The prey was reared in 250 ml plastic containers with screw caps fitted with meshed holes for ventilation. The rearing medium consisted of a mixture of wheat bran, dry yeast, and icing sugar. The cultures were maintained in a controlled environment at 25 °C ± 1 °C, 80% ± 5% relative humidity (RH), and a 16:8 h light: dark photoperiod at the Manaaki Whenua—Landcare Research, St Johns, Auckland, New Zealand.

The initial population of *E. sexmaculatus* used as prey in this study was collected on avocado leaves from an orchard in Katikati, Bay of Plenty, New Zealand (−37.5307815, 175.9171147) and maintained on the leaves of avocado seedlings (c. 1 m in height) at 25 °C ± 3 °C, 40% ± 5% RH, under natural daylight. The avocado leaves were gently washed with water before introducing *E. sexmaculatus*. To prevent contamination from other predators, a thick layer of Vaseline was applied to the base of the avocado seedlings.

#### Predatory Mites

Both predatory mite species used in this study were originally collected on avocado and plum leaves from Rototuna, Hamilton, New Zealand in March 2024. The 2 predator species were reared using identical rearing set-ups: a petri dish was placed on top of a plastic sheet, which was then placed on a slightly larger piece of sponge (see Wang et al. 2024 for details). The sponge was soaked in water-filled containers. Mites had access to water but were unable to escape from their cultures, preventing contamination. The wheat bran mixture with *C. lactis* was added to the rearing set-ups of predatory mites. Four square plastic sheets (c. 10 mm long), folded twice, were added to each culture to provide refuge for the predatory mites.

For the experiments, similarly aged cohorts of both predatory species were established by transferring eggs (<24 h old) into new cultures with ad libitum supply of *C. lactis*. The eggs of predatory species were collected by placing sewing threads (c. 15 mm long) + into the main cultures overnight (Zhang and Zhang 2022). All adults of both species used in the experiments were young adults (less than 7 d after reaching maturity).

### Experimental Arenas

For the experimental arenas used in the pretest starvation treatment, the cell consisted of 2 transparent plexiglass slides (L38 × W25 × H2 mm), and the aperture in this cell was conical (a diameter of 6 mm at the top, 10 mm at the bottom). A black plastic sheet was placed between 2 plexiglass slides, then 4 layers of filter paper were placed under the plastic sheet for adding water, and 7 holes were pierced in the plastic sheet using a size 3 insect pin to allow access to water for the predators (see Wang et al. 2024 for details). The entire cell assembly was secured using a pair of metal clips. A layer of food wrap was used to seal the top of the cylindrical hole. Seven evenly spaced holes were pierced into the wrap using a size 0 insect pin to allow ventilation.

The arena used in predation bioassays was similar to the cell described above but used transparent plexiglass square slides (L38 × W38 × H3 mm) and a cylindrical aperture with a diameter of 15 mm in the middle of the upper slide, which was the arena space for testing. A fresh avocado leaf disc (lower leaf surface upward) with a diameter of 20 mm was used instead of the black plastic sheet, and the freshness of this leaf disc ensured by adding water to the filter paper.

### Experiment 1: No-choice Tests

Each no-choice test lasted for 24 h and occurred in the same controlled laboratory environment as the predator cultures. Each predatory species was replicated 10 times for each prey stage treatment. Before trials began, an adult (confirmed gravid) female (<7 d old) was transferred into an empty arena and starved for 24 h to standardize the predator’s hunger level; water was added to the 4 layers of filter paper before the test. Then, before the end of starvation treatment, one of 4 prey stage treatments was applied as follows: (i) 40 eggs, (ii) 40 larvae, (iii) 40 nymphs, or (iv) 20 adult females. Starved predators were placed in the center of the test arena and the top of the cell was immediately sealed with the piece of food wrap, and water was added to 4 layers of filter paper. The following data were recorded: (i) number of prey consumed; (ii) number of eggs laid by the predator; (iii) number of new eggs laid by female adult prey in a treatment group (to compare the differences in possible non-feeding effects on *E. sexmaculatus* oviposition due to *A. limonicus* and *A. herbicolus*); and (iv) the status of eggs in the egg treatment group (ie possible partial consumption, referring to an egg which was destroyed but not completely consumed).

### Experiment 2: Choice Tests

The choice tests used the same set-up as the no-choice tests and lasted for 24 h, but the prey treatments included a mixture of 10 eggs, 10 larvae, 10 nymphs, and 10 female adults in each experimental arena. As in the no-choice test, there were 10 replicates for each predator species for each treatment. At the end of test, the same information as the no-choice test was recorded.

### Statistical Analysis

The software R, Version 4.4.0 (R Core Team 2024) was used for statistical analysis in this study. The results were summarized as means and standard errors of the mean (SEMs). Due to the non-normal distribution of the whole data recorded, aligned Rank Transform (ART) analysis of variance (ANOVA) in the package *ARTool* (Kay et al. 2021) was used to compare the number of prey consumed by different treatment groups, the number of eggs laid by predators, and the number of eggs laid by prey female adults. A Wilcoxon rank-sum test was used to compare all the numerical differences in this study of the 2 predators under the same treatment.

The package *selectapref* (Richardson 2020) was used to generate the Manly’s preference index (Manly 1974) in the choice test (Experiment 2), which was used to assess the preference of *A. limonicus* and *A. herbicolus* for different prey stages. Using the preference index for eggs as an example, the formula is given below:


$$\begin{array}{l} \alpha =\frac{\log\frac{e_{1}}{A_{1}}}{\log\frac{e_{1}}{A_{1}}+\log\frac{e_{2}}{A_{2}}+\log\frac{e_{3}}{A_{3}}+\log\frac{e_{4}}{A_{4}}} \end{array}$$

where α refers to the preference index; *e*_1_, *e*_2_, *e*_3,_ and *e*_4_ refer to the numbers of consumed *E. sexmaculatus* eggs, larvae, nymphs and female adults, respectively; *A*_1_, *A*_2_, *A*_3,_ and *A*_4_ refer to the initial numbers of *E. sexmaculatus* placed in the cell at the start of each test (in this study, *A*_1_, *A*_2_, *A*_3_, and *A*_4_ each = 10). A higher α value represents the higher preference of the predator for that prey’s life stage. The package *ggplot2* (Wickham 2016) was used for graphical representation. Statistical significance was set at *P* < 0.05.

## Results

### Experiment 1: No-choice Test

**Fig. 1.**
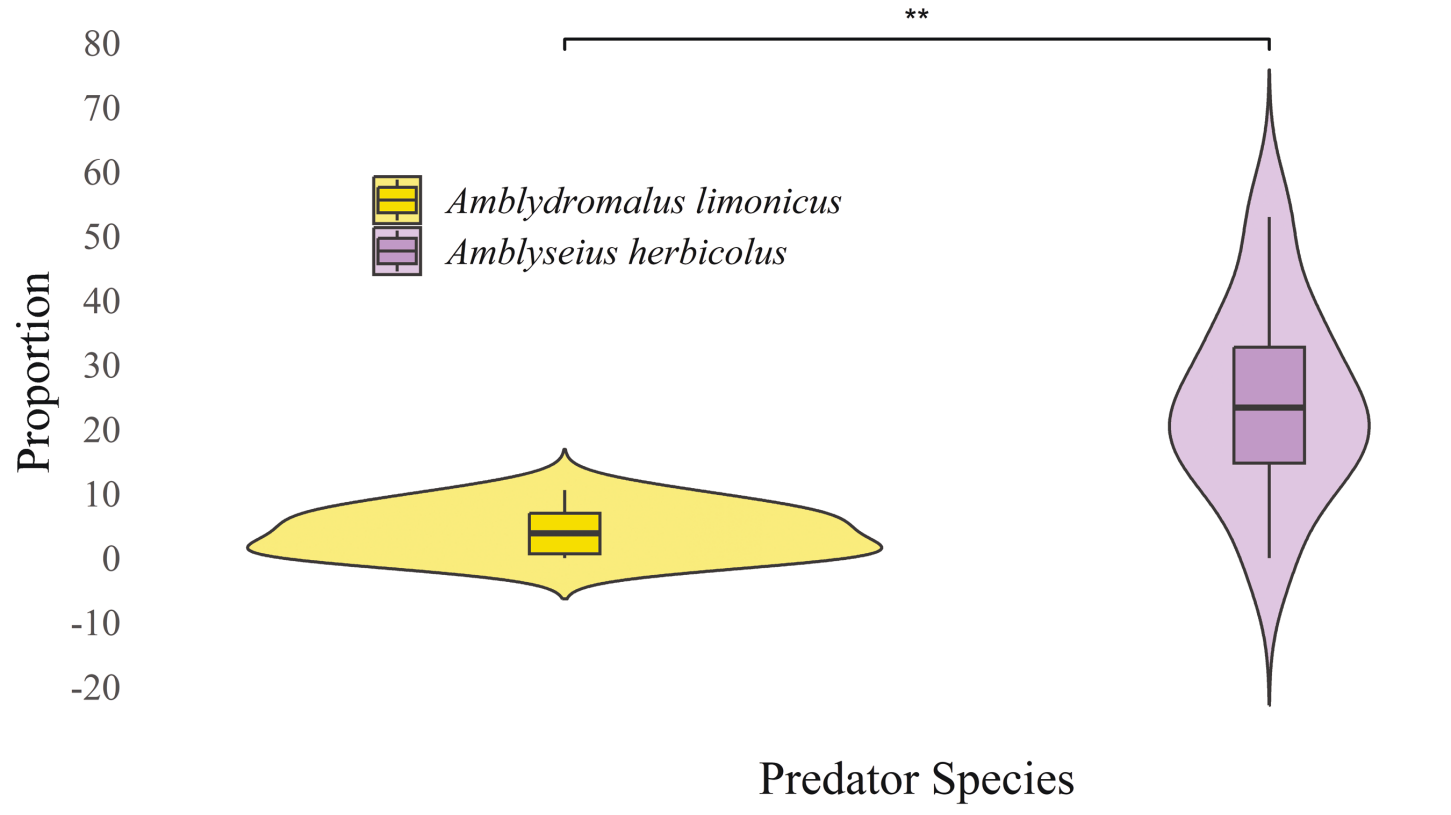
Experiment 1 (no-choice) egg treatment group data showing the proportion of prey eggs partially consumed by *Amblydromalus limonicus* and *Amblyseius herbicolus* out of all prey eggs consumed over a 24-h period. Asterisks denote the significant differences between two predator species (Wilcoxon rank-sum test: **P < 0.01). The “cloud” represents the kernel density estimation (KDE), showing the smoothed distribution of the data. The width of the “cloud” reflects the density of the data (the wider the clouds, the more possible data point estimates, or *vice versa*).

Consumption of each of the 4 prey stages (ie eggs, larvae, nymphs, and adults) was observed in both predator species. Overall, adult *A. limonicus* females consumed significantly more prey than adult *A. herbicolus* females (ART ANOVA: *F*_1,72_ = 93.92, *P *< 0.001; **Table 1**). Prey consumption by both predator species was significantly affected by the prey life stage (*F*_3,72_ = 186.71, *P *< 0.001), with the highest consumption observed for eggs (**Table 1**). There was a significant interaction between predator species and prey life stage (*F*_3,72_ = 7.42, *P *< 0.001): the difference in prey consumption between *A. limonicus* and *A. herbicolus* was more pronounced for the earlier prey life stages than later stages (**Table 1**). Specially, the comparison of the results of 2 treatment groups of predators feeding on prey eggs showed that some of the eggs were not completely consumed by both predators, and the proportion of eggs partially consumed by *A. limonicus* was significantly lower than that for *A. herbicolus* (**Fig. 1**). Additionally, in the treatment group involving 20 adult *E. sexmaculatus* females, there were a small number of replicates, *n* = 3 (*A. limonicus*) and *n* = 2 (*A. herbicolus*), in which the predators completely sucked up one newly laid *E. sexmaculatus* egg.

**Table 1.** Prey consumption (mean ± SEM, min–max) by adult female *Amblydromalus limonicus* and *Amblyseius herbicolus* feeding on different life stages of the prey *Eotetranychus sexmaculatus* in the no-choice test.

Prey	No. prey	*Amblydromalus limonicus*	*Amblyseius herbicolus*
Eggs	40	38.8 ± 0.3 (37–40)^a^**	28.3 ± 2.2 (16–36)^a^**
Larvae	40	33.3 ± 1.4 (26–40)^b^**	20.8 ± 1.6 (14–28)^b^**
Nymphs	40	13.4 ± 0.9 (8–18)^c^*	9.2 ± 0.8 (5–14)^c^*
Female adults	20	4.8 ± 0.6 (2–8)^d^**	1.6 ± 0.2 (1–3)^d^**

^abcd^Means followed by different letters are significantly different according to ART ANOVA pairwise comparisons. Asterisks denote significant differences in prey consumption within the same prey life stage between two species of predatory mites (Wilcoxon rank-sum test:

** *P *< 0.01;

* *P* < 0.05).

When feeding on *E. sexmaculatus* adult females, *A. limonicus* consumed more individuals than *A. herbicolus* (**Table 1**). However, the difference between 2 predators’ interference with oviposition rates of the remaining adult *E. sexmaculatus* females during tests was not significant (*F*_1,18_ = 0.56, *P* = 0.464): the number of eggs laid by adult *E. sexmaculatus* females in the 2 treatment groups were 0.94 ± 0.10 eggs in *A. limonicus* groups; and 1.10 ± 0.12 eggs in *A. herbicolus* groups.

### Experiment 2: Choice Test

When adult females of *A. limonicus* and *A. herbicolus* were given a mixture of prey life stages, while the numbers of prey eggs/larvae consumed by 2 predator species was not significantly different (**Table 2**), the difference in overall consumption was significant (*F*_1,72_ = 15.56, *P* < 0.001). The consumption of prey at different life stages by both *A. limonicus* and *A. herbicolus* was highly significant (*F*_3,72_ = 94.12, *P* < 0.001), with higher consumption of prey at earlier life stages (**Table 2**). The interaction between predator species and prey life stage was also significant (*F*_3,72_ = 7.62, *P* = 0.012); this was reflected in *A. herbicolus*’ stronger preference for eggs than *A. limonicus* (**Table 2**).

**Table 2.** Prey consumption (mean ± SEM, min–max) by adult female *Amblydromalus limonicus* and *Amblyseius herbicolus* feeding on different life stages of the prey *Eotetranychus sexmaculatus* in the choice test.

Prey	No. prey	*Amblydromalus limonicus*	*Amblyseius herbicolus*
Eggs	10	7.6 ± 0.6 (4–10)^a^	9.0 ± 0.4 (7–10)^a^
Larvae	10	4.9 ± 0.3 (3–6)^b^	3.2 ± 0.6 (0–6)^b^
Nymphs	10	3.8 ± 0.4 (2–6)^c^**	1.1 ± 0.5 (0–5)^c^**
Female adults	10	1.4 ± 0.2 (0–2)^d^**	0.3 ± 0.1 (0–1)^c^**

^abcd^Means followed by different letters are significantly different according to ART ANOVA pairwise comparisons. Asterisks denote significant differences between prey consumption for each species within the same prey life stage (Wilcoxon rank-sum test:

** *P *< 0.01).

### Oviposition Rates in Choice and no-choice Bioassays

**Fig. 2.**
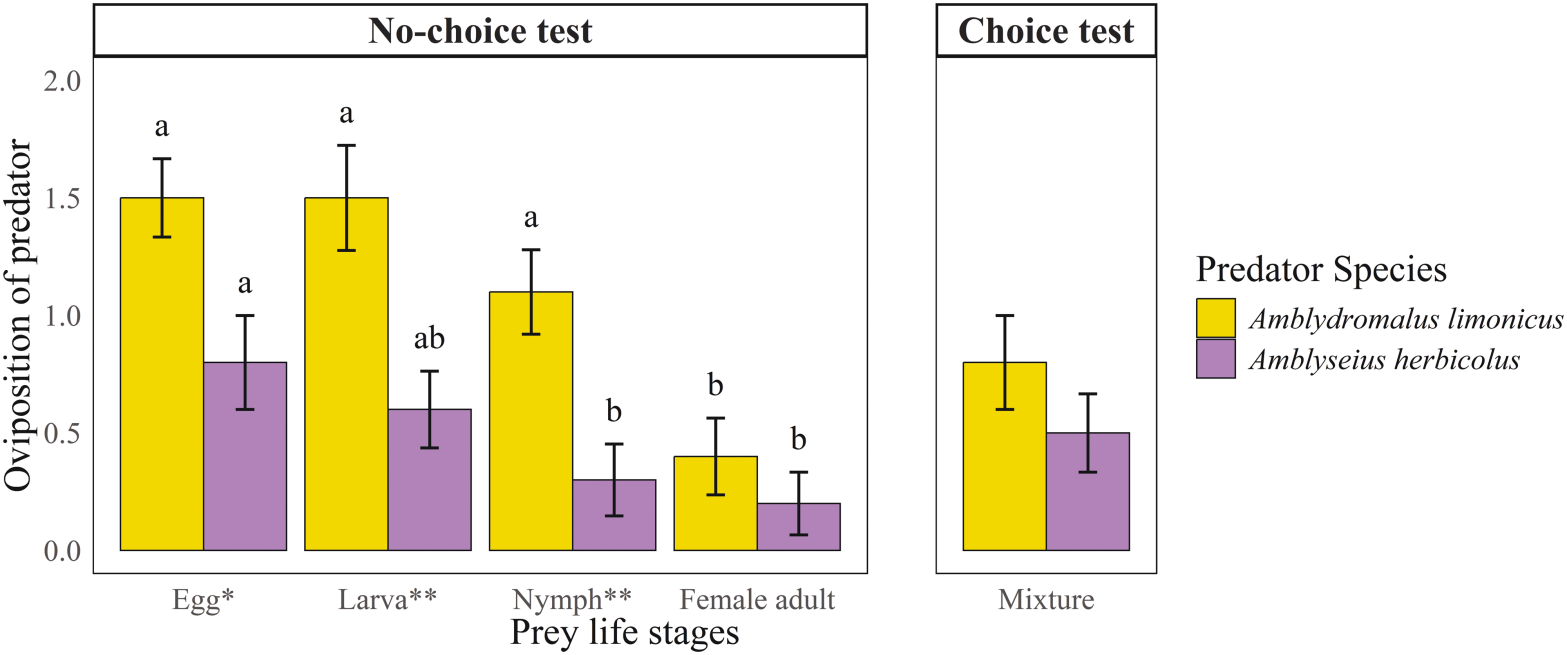
The number (Mean ± SEM) of eggs laid by *Amblydromalus limonicus* and *Amblyseius herbicolus* in the arena during no-choice (left) and choice (right) tests. Asterisks denote significant differences in oviposition rate within the same prey life stage between two species of predatory mites (Wilcoxon rank-sum test: ** *P *< 0.01; * *P* < 0.05). ^ab^ Means ART ANOVA pairwise comparisons in oviposition rate within the same species of predatory mites, different letters denote significant differences.

*Amblydromalus limonicus* laid significantly more eggs than *A. herbicolus* (*F*_1,72_ = 22.90; *P* < 0.001) in treatments of all 4 prey life stages in both the choice and no-choice experiments (**Fig. 2**). The number of eggs laid by *A. limonicus* and *A. herbicolus* in the experimental arena was significantly affected by the life stage of the prey (*F*_3,72_ = 10.77; *P* < 0.001), with this number gradually decreasing as the life stage of the prey increased (**Fig. 2**). However, there were no significant interactions between predator species and prey stage (*F*_3,72_ = 1.26; *P* = 0.296).

Most females of both predator species laid eggs when feeding on a mixture of prey life stages, but the difference in the number of eggs laid was non-significant between the 2 predator species (**Fig. 2**). As in the no-choice tests, there was no significant difference in the oviposition rates by the remaining adult *E. sexmaculatus* females in the 2 treatment groups (*F*_1,18_ = 1.05, *P* = 0.319): (1.10 ± 0.11 eggs in *A. limonicus* groups; 0.93 ± 0.11 eggs in *A. herbicolus* groups).

**Fig. 3.**
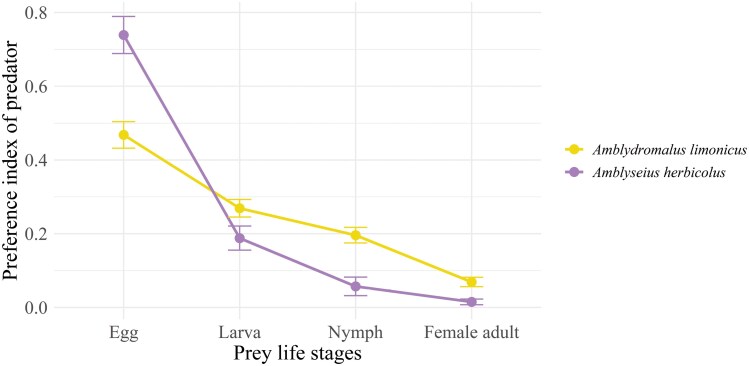
Choice test (Experiment 2): Manly’s preference index curves for adult females of *Amblydromalus limonicus* and *Amblyseius herbicolus* consuming different life stages of the prey *Eotetranychus sexmaculatus*. Bars show mean ± SEM values.

In this choice test, the calculated Manly’s preference index indicated a preference for earlier prey life stage by both *A. limonicus* and *A. herbicolus* (*F*_3,72_ = 105.43; *P* < 0.001; **Fig. 3**). There was a significant interaction between predator species and prey life stage (*F*_3,72_ = 19.27; *P* < 0.001), reflected in the stronger preference for eggs shown by *A. herbicolus* compared to *A. limonicus* (**Fig. 3**).

## Discussion

In this study, we demonstrated for the first time that both *A. limonicus* and *A. herbicolus* can feed on the eggs, larvae, nymphs, and adult females of *E. sexmaculatus* on avocado leaf discs and that both species preferred earlier life stages of *E. sexmaculatus.* We also showed that both predator species could reproduce when feeding on different life stages of *E. sexmaculatus*. These results provide the first laboratory evidence that these 2 predatory species have the potential for use in biological control against *E. sexmaculatus*.

### Predation

In the no-choice tests (Experiment 1), we observed that *A. limonicus* consumed significantly more prey than *A. herbicolus* across all *E, sexmaculatus* life stages (**Table 1**). Within the limits of the amount of prey being tested, young female *A. limonicus* adults were able to consume almost all the 40 *E. sexmaculatus* eggs or most of the 40 *E. sexmaculatus* larvae within 24 h, although the consumption might be greater if more prey eggs/larvae were provided. In the egg treatment, we observed that some prey eggs were partially consumed by *A. limonicus* or *A. herbicolus*, with such eggs being more frequently observed when *A. herbicolus* was the predator. This could indicate that the eggs of *E. sexmaculatus* might not provide sufficient nutrients to *A. herbicolus*. However, partially consumed eggs cannot hatch, so *A. herbicolus* still had some biological control effect on these *E. sexmaculatus* eggs. In phytoseiid-spider mites’ predator–prey interactions, the rate of prey consumption usually decreases with increasing life stage, although sometimes larval consumption is higher than egg consumption (Ali et al. 2011). In both *E. sexmaculatus* nymph and female adult treatment groups, the consumption rate by *A. limonicus* and *A. herbicolus* seemed to decrease more in comparison with egg and larva treatment groups (**Table 1**). Additionally, as *E. sexmaculatus* grows in size with subsequent life stages (McGregor 1950), the webs produced by *E. sexmaculatus* nymphs and adults seemed to hinder and entangle *A. limonicus* and *A. herbicolus*, reducing their rates of predation (Pers, observation of Y. Yang). This is consistent with previous reports of these 2 predators being hampered by webs from other spider mites (Sabelis and Bakker 1992, Wang et al. 2024). Although the prey in the female adult treatment groups laid some new eggs, the predators rarely consumed these eggs for food. This may also be because the newly laid eggs were protected by webs, making it difficult for predators to reach them.

In the choice test (Experiment 2), the predation efficiency of *A. limonicus* appeared to be similar to that of the no-choice test over the same test time (24 h), but the efficiency of *A. herbicolus* seemed to decrease (**Table 2**). Compared to other choice tests for spider mites, the predation efficiency of *A. limonicus* and *A. herbicolus* in this study was obviously lower than that reported in Hao et al. (2021). There are 4 possible explanations for this difference. (i) Differences exist in predation efficiency among different genera of predators. (ii) The host influences on the performance of *E. sexmaculatus* may be different on rubber *versus* avocado leaves (Lu et al. 2016, Liang et al. 2020). (iii) The 3-dimensional and relatively closed set-ups of the test arena simulated in this study might strengthen the effects of *E. sexmaculatus* webs in limiting predator mobility (Sabelis and Bakker 1992, Shimoda et al. 2010). (iv) Compared with the study of Hao et al. (2021), the higher prey density provided by this study might interfere with the predator’s hunting efficiency (Reis et al. 2003).

### Prey Preference

In the choice test (Experiment 2), *A. limonicus* and *A. herbicolus* clearly favored earlier prey stages, which is similar to the results reported for *A. swirskii* (Hao et al. 2021). The preference was more marked in *A. herbicolus*. Many studies have investigated the prey preferences of species in the family Phytoseiidae, and variable nutritional value of the different predation strategies in the face of different spider mite prey and thus show different predation preferences (Blackwood et al. 2001, Xiao and Fadamiro 2010, Jyothis and Ramani 2019, 2024). Possible factors include the probability of encountering prey (Sabelis 1990), how easy it is to hold onto the prey (Furuichi et al. 2005), and the availability of nutritional value at different stages of the prey’s life (Sabelis 1990, Blackwood et al. 2001, Xiao and Fadamiro 2010). Different predation preferences may also be related to the degree to which predators are specialized for hunting different prey (Blackwood et al. 2001, McMurtry et al. 2013). The possible interference from the *E. sexmaculatus* webs with predator behavior may have changed the predation preferences of *A. limonicus* or *A. herbicolus* in this study, consistent with several previous studies (Sabelis and Bakker 1992, Shimoda et al. 2010, McMurtry et al. 2013). In no replicate in the choice test, did we find even one newly laid egg consumed by predators, but the eggs which were artificially set without the protection of webs were heavily consumed. Active prey could leave the protection of the webs and be consumed; thus, the number of prey remaining seems to reflect the ability of prey to detect the predator and hide in the protection of the webs. Under this assumption, *A. herbicolus* might be less successful in capturing and consuming *E. sexmaculatus* than *A. limonicus*, resulting in *A. herbicolus* having to consume more artificially set eggs for survival and showing more extreme preferences.

### Oviposition Rate

In this study, young female predators in the no-choice experiments laid an average of 1.5 (*A. limonicus*) and 0.8 (*A. herbicolus*) eggs, respectively, when provided with the eggs of *E. sexmaculatus*, but the rate of oviposition decreased with increasing prey life stage (**Fig. 2**). In the choice experiments, young female predators laid an average of 0.8 (*A. limonicus*) and 0.5 (*A. herbicolus*) eggs, respectively (**Fig. 2**). Our follow-up observations showed that these eggs were able to hatch. Oviposition rates reflect the ability of a predator to reproduce using the nutrients in its prey, and it can be used to predict whether artificially released predators can control *E. sexmaculatus* prey effectively and sustainably in the avocado leaf habitat. Compared to the other predation tests, the oviposition rate of *A. limonicus* consuming *E. sexmaculatus* in our study was similar to that of *A. limonicus* when feeding on other suitable prey species in other laboratory studies (Davidson et al. 2016, Patel and Zhang 2017, Lam et al. 2019), which seems to indicate that *A. limonicus* can effectively use and convert the nutrients of *E. sexmaculatus* into offspring. However, the oviposition rate of *A. herbicolus* consuming *E. sexmaculatus* was significantly lower than that of *A. herbicolus* when consuming other suitable prey species in other laboratory studies (Lam et al. 2019, Liu et al. 2024). This seems to suggest that *A. herbicolus* is not well suited to consuming *E. sexmaculatus* as prey for total nutrition alone. This makes it is uncertain whether *A. herbicolus*, if released, would choose to consume *E. sexmaculatus*, other prey, or possibly even *A. herbicolus* larvae (Liu et al. 2023) as the food source.

A further consideration is that our review of previous studies on *A. limonicus* found that the daily oviposition rate reported in studies using the same prey in long-term rearing was significantly higher than the reported single-day oviposition rate of studies simulating artificial release (McMurtry and Scriven 1965, Samaras et al. 2015). This was consistent with the interference of the previous prey on subsequent short-term oviposition rate, as reported in Sabelis (1990).

### Predator Lifestyle

In this study, *A. limonicus* and *A. herbicolus*, 2 biological control agents commonly used against thrips, psyllids, and whiteflies, showed statistically significantly different results against the spider mite *E. sexmaculatus*. Differences in lifestyle may explain this: *A. limonicus*, a type III-b predator, prefers glabrous leaves as a habitat, while *A. herbicolus*, a type III-c predator, prefers confined space on dicotyledonous plants (McMurtry et al. 2013). Therefore, the less hairy and more open avocado leaves may be more suitable for *A. limonicus*. This is also consistent with our observations when collecting predators. The location from where we collected *A. limonicus* and *A. herbicolus* included neighboring avocado and plum trees: most *A. limonicus* were observed on avocado leaves, while most *A. herbicolus* observed on plum leaves.

### Limitations of Current Study and Suggestion for Future Studies

This study was a laboratory simulation of predator release in the field, and, as such, certain factors confound interpretation. (i) The test arena was more enclosed than the avocado leaves, which could have led to excessive interference from predators with the 3-dimensional webbing structure; (ii) The experimental set-up involved simpler interspecific interactions than natural niches, and predators may prefer to consume prey other than *E. sexmaculatus* (eg *A. herbicolus* may prefer thrips) after release in the field. (iii) The higher density of prey in our laboratory study probably interfered with the predator’s living space. Expanded greenhouse experiments or field experiments should then be conducted as follow ups to provide better evidence of whether these predators can act as effective biological control agents.

Overall, this study demonstrates that *A. limonicus* and *A. herbicolus* can consume *E. sexmaculatus* at various life stages on avocado leaves. Of the 2 predators, *A. limonicus*, which has been commercialized, can be more quickly and easily applied to actual avocado production. However, the webs of *E. sexmaculatus* may be a key factor in reducing the predation efficiency of both predators. Future studies will need to consider the extent to which the webs hinder *A. limonicus* or *A. herbicolus*, and whether both of these predators can be used as practical biological control agents in integrated pest management programs.

## References

[CIT0001] Ali M , Naif AA, Huang D. 2011. Prey consumption and functional response of a phytoseiid predator, *Neoseiulus womersleyi*, feeding on spider mite, *Tetranychus macfarlanei*. J. Insect Sci. 11:167. https://doi.org/10.1093/jis/11.1.167

[CIT0002] Assouguem A , Kara M, Mechchate H, et al 2022. Current situation of *Tetranychus urticae* (Acari: Tetranychidae) in Northern Africa: the sustainable control methods and priorities for future research. Sustainability. 14:2395. https://doi.org/10.3390/su14042395

[CIT0003] Ayala Silva T , Ledesma N. 2014. Avocado history, biodiversity and production. In: Nandwani D editor. Sustainable horticultural systems. sustainable development and biodiversity. Vol 2. Springer. p. 157–205.

[CIT0004] Babendreier D. 2007. Pros and cons of biological control. In: Nentwig W editor. Biol. invasions. Springer. p. 403–418.

[CIT0005] Blackwood J , Schausberger P, Croft B. 2001. Prey-stage preference in generalist and specialist phytoseiid mites (Acari: Phytoseiidae) when offered *Tetranychus urticae* (Acari: Tetranychidae) eggs and larvae. Environ. Entomol. 30:1103–1111. https://doi.org/10.1603/0046-225X-30.6.1103

[CIT0006] Bragard C , Baptista P, Chatzivassiliou E, et al; EFSA Panel on Plant Health (EFSA PLH Panel). 2023. Pest categorisation of *Eotetranychus sexmaculatus*. EFSA J. 21:e07898. https://doi.org/10.2903/j.efsa.2023.7898 37009445 PMC10052452

[CIT0007] Caceres S , Childers CC. 1991. Biology and life tables of *Galendromus helveolus* (Acari: Phytoseiidae) on Florida citrus. Environ. Entomol. 20:224–229. https://doi.org/10.1093/ee/20.1.224

[CIT0008] Cardoso AC , Marcossi I, Fonseca MM, et al 2024. A predatory mite as potential biological control agent of *Bemisia tabaci* on tomato plants. J. Pest Sci. 1–13. https://doi.org/10.1007/s10340-024-01809-7

[CIT0009] Chen J , Zheng L, Ye Z, et al 2023. Evaluation of the predatory mite *Neoseiulus barkeri* against spider mites damaging rubber trees. Insects. 14:648. https://doi.org/10.3390/insects14070648 37504654 PMC10380992

[CIT0010] Cuthbertson AG. 2014. The feeding rate of predatory mites on life stages of *Bemisia tabaci* Mediterranean species. Insects. 5:609–614. https://doi.org/10.3390/insects5030609 26462828 PMC4592588

[CIT0011] Davidson MM , Nielsen M-C, Butler RC, et al 2016. Prey consumption and survival of the predatory mite, *Amblydromalus limonicus*, on different prey and host plants. Biocontrol Sci. Technol. 26:722–726. https://doi.org/10.1080/09583157.2016.1143916

[CIT0012] Erichsen C , Schoeman A. 1992. Economic losses due to insect pests on avocado fruit in the Nelspruit/Hazyview region of South Africa during 1991. South Afr. Avocado Growers’ Assoc. Yearbook 1992. 15:49–54. http://avocadosource.com/Journals/SAAGA/SAAGA_1992/SAAGA_1992_PG_49-54.pdf

[CIT0013] Froud K , Stevens P, Machin T et al 2002. Efficacy of new pesticides against sixspotted mite *Eotetranychus sexmaculatus* (Riley)(Acari: Tetranychidae) on avocados. *NZ Avocado Growers’ Association Annual Research Report Volume 2, 2002*. https://citeseerx.ist.psu.edu/document?repid=rep1&type=pdf&doi=88039761b7c9f69c476006cfc599eb7a0c9bb6cd

[CIT0014] Furuichi H , Oku K, Yano S, et al 2005. Why does the predatory mite *Neoseiulus womersleyi* Schicha (Acari: Phytoseiidae) prefer spider mite eggs to adults? Appl. Entomol. Zool. 40:675–678. https://doi.org/10.1303/aez.2005.675

[CIT0015] García JSA , Hurtado-Salazar A, Ceballos-Aguirre N. 2021. Current overview of Hass avocado in Colombia. Challenges and opportunities: a review. Ciência Rural. 51:e20200903. https://doi.org/10.1590/0103-8478cr20200903

[CIT0016] Hajek AE , Hurley BP, Kenis M, et al 2016. Exotic biological control agents: a solution or contribution to arthropod invasions? Biol. Invasions. 18:953–969. https://doi.org/10.1007/s10530-016-1075-8

[CIT0017] Hao H , Li P, Xu T, et al 2021. Preliminary evaluation of the control effect of two predatory mite species on *Eotetranychus sexmaculatus* in rubber trees in Hainan Province, China. Syst. Appl. Acarol. 26:2287–2296. https://doi.org/10.11158/saa.26.12.7

[CIT0019] Hoddle MS , Morse JG. 2012. The persea mite invasion into California: history, biology, management and current status. California Avocado Soc. 2012 Yearbook. 95:107–137. https://www.avocadosource.com/cas_yearbooks/cas_95_2012/cas_2012_v95_pg_107-137.pdf

[CIT0018] Hoddle MS , Jetter KM, Morse JG. 2003. The economic impact of *Scirtothrips perseae* Nakahara (Thysanoptera: Thripidae) on California avocado production. Crop. Prot. 22:485–493. https://doi.org/10.1016/s0261-2194(02)00199-0

[CIT0020] Jamieson L , Stevens P. 2007. Development rates longevity and fecundity of sixspotted mite (*Eotetranychus sexmaculatus*) at constant temperatures. N. Z. Plant Prot. 60:72–77. https://doi.org/10.30843/nzpp.2007.60.4612

[CIT0021] Jorge SJ , Rueda-Ramírez D, de Moraes G. 2021. Predation capacity of phytoseiid mites (Mesostigmata: Phytoseiidae) from Brazil on eggs of *Diaphorina citri* (Hemiptera: Liviidae). Phytoparasitica. 49:603–611. https://doi.org/10.1007/s12600-021-00898-9

[CIT0022] Jyothis D , Ramani N. 2019. Evaluation of prey stage preference of the predatory mite *Neoseiulus longispinosus* (Evans) on the spider mite pest *Tetranychus neocaledonicus* (André)(Acari: Phytoseiidae, Tetranychidae). Acarologia. 59:484–491. https://doi.org/10.24349/acarologia/20194347

[CIT0023] Jyothis D , Ramani N. 2024. Prey stage preference of *Amblyseius paraaerialis* (Acari: Phytoseiidae) on varied life stages of the spider mites *Tetranychus urticae, Tetranychus macfarlanei* and *Oligonychus biharensis* (Acari: Tetranychidae) and exploring the mass rearing possibilities of this predatory mite on alternative diets. Exp. Appl. Acarol. 92:385–401. https://doi.org/10.1007/s10493-023-00899-9 38478140

[CIT0024] Kay M , Elkin L, Higgins J et al 2021. ARTool: aligned rank transform for nonparametric factorial ANOVAs. R package 0.11.1. https://github.com/mjskay/ARTool

[CIT0025] Knapp M , van Houten Y, Hoogerbrugge H, et al 2013. *Amblydromalus limonicus* (Acari: Phytoseiidae) as a biocontrol agent: literature review and new findings. Acarologia. 53:191–202. https://doi.org/10.1051/acarologia/20132088

[CIT0026] Knapp M , van Houten Y, van Baal E, et al 2018. Use of predatory mites in commercial biocontrol: current status and future prospects. Acarologia. 58:72–82. https://doi.org/10.24349/acarologia/20184275

[CIT0027] Lam W , Paynter Q, Zhang Z-Q. 2019. Predation, prey preference and reproduction of predatory mites *Amblydromalus limonicus* (Garman), *Amblyseius herbicolus* (Chant) and *Neoseiulus cucumeris* (Oudemans) (Mesostigmata: Phytoseiidae) on immature *Sericothrips staphylinus* Haliday (Thysanoptera: Thripidae), a biocontrol agent of gorse. Syst. Appl. Acarol. 24:508–519. https://doi.org/10.11158/saa.24.3.14

[CIT0028] Learmonth S. 2019. Pest status and management of SSM (*Eotetranychus sexmaculatus*) in WA Avocado Orchards. Final Report Project AV15012. Hort Innovation, Sydney, Australia. https://www.horticulture.com.au/globalassets/laserfiche/assets/project-reports/av15012/av15012---final-report-complete.pdf

[CIT0029] Lee MH , Zhang Z-Q. 2018. Assessing the augmentation of *Amblydromalus limonicus* with the supplementation of pollen, thread, and substrates to combat greenhouse whitefly populations. Sci. Rep. 8:12189. https://doi.org/10.1038/s41598-018-30018-3 30111848 PMC6093862

[CIT0030] Liang X , Chen Q, Wu C, et al 2020. Reference gene validation in *Eotetranychus sexmaculatus* (Acari: Tetranychidae) feeding on mite-susceptible and mite-resistant rubber tree germplasms. Exp. Appl. Acarol. 82:211–228. https://doi.org/10.1007/s10493-020-00542-x 32886259

[CIT0031] Liu J-F , Zhang Z-Q. 2017. Development, survival and reproduction of a New Zealand strain of *Amblydromalus limonicus* (Acari: Phytoseiidae) on *Typha orientalis* pollen, *Ephestia kuehniella* eggs, and an artificial diet. Int. J. Acarol. 43:153–159. https://doi.org/10.1080/01647954.2016.1273972

[CIT0032] Liu JF , Zhang ZQ, Beggs JR, et al 2019. Provisioning predatory mites with entomopathogenic fungi or pollen improves biological control of a greenhouse psyllid pest. Pest Manag. Sci. 75:3200–3209. https://doi.org/10.1002/ps.5438 30957393

[CIT0033] Liu W , Zhang K, Zhang Z-Q. 2023. Larval feeding types shape the predation aggression of predatory mites in both intraspecific and interspecific encounters. Syst. Appl. Acarol. 28:1272–1282. https://doi.org/10.11158/saa.28.7.6

[CIT0034] Liu Z , Zhang K, Zhang Z-Q. 2024. Unintended consequences: the adverse effects of royal jelly supplementation in the predatory mite *Amblyseius herbicolus* Chant (Acari: Phytoseiidae). Syst. Appl. Acarol. 29:335–345. https://doi.org/10.11158/saa.29.2.12

[CIT0035] Logan D , Barraclough E, Poulton J, et al 2023. Six-spotted mite and its predators on sprayed and unsprayed ‘Hass’ avocado trees. N. Z. J. Crop Hortic. Sci. 51:198–211. https://doi.org/10.1080/01140671.2021.2020852

[CIT0036] Lu F , Chen Z, Lu H, et al 2016. Effects of resistant and susceptible rubber germplasms on development, reproduction and protective enzyme activities of *Eotetranychus sexmaculatus* (Acari: Tetranychidae). Exp. Appl. Acarol. 69:427–443. https://doi.org/10.1007/s10493-016-0049-y 27188510

[CIT0037] Majid D , Dar B, Parveen S, et al 2020. Avocado. In: Nayik GA, Gull A, editors. Antioxidants in fruits: properties and health benefits. Springer. p. 103–123.

[CIT0038] Manly B. 1974. A model for certain types of selection experiments. Biometrics 30:281–294. https://doi.org/10.2307/2529649

[CIT0039] McGregor EA. 1950. Mites of the family Tetranychidae. Am. Midl. Nat. 44:257–420. https://doi.org/10.2307/2421963

[CIT0040] McMurtry J. 1989. 12.1 Utilizing natural enemies to control pest mites on citrus and avocado in California, USA. In: Channabasavanna GP, Viraktamath CA editors. Progress in acarology. VII international congress of acarology, Bangalore, Aug 1986. Vol 2. Oxford and IBH Publishing. p. 325–336.

[CIT0041] McMurtry J , Scriven G. 1965. Life-history studies of *Amblyseius limonicus*, with comparative observations on *Amblyseius hibisci* (Acarina: Phytoseiidae). Ann. Entomol. Soc. Am. 58:106–111. https://doi.org/10.1093/aesa/58.1.106

[CIT0042] McMurtry J , Scriven G. 1971. Predation by *Amblyseius limonicus* on *Oligonychus punicae* (Acarina): Effects of initial predator-prey ratios and prey distribution. Ann. Entomol. Soc. Am. 64:219–224. https://doi.org/10.1093/aesa/64.1.219

[CIT0043] McMurtry JA , De Moraes GJ, Sourassou NF. 2013. Revision of the lifestyles of phytoseiid mites (Acari: Phytoseiidae) and implications for biological control strategies. Syst. Appl. Acarol. 18:297–320. https://doi.org/10.11158/saa.18.4.1.

[CIT0044] McMurtry JA , Sourassou NF, Demite PR. 2015. The Phytoseiidae (Acari: Mesostigmata) as biological control agents. In: Carrillo D, de Moraes GJ, Peña JE editors. Prospects for biological control of plant feeding mites and other harmful organisms. Springer. p. 133–149.

[CIT0045] Medd NC , GreatRex RM. 2014. An evaluation of three predatory mite species for the control of greenhouse whitefly (*Trialeurodes vaporariorum*). Pest Manag. Sci. 70:1492–1496. https://doi.org/10.1002/ps.3794 24706366

[CIT0046] Mouratidis A , Marrero-Díaz E, Sánchez-Álvarez B, et al 2023. Preventive releases of phytoseiid and anthocorid predators provided with supplemental food successfully control Scirtothrips in strawberry. BioControl. 68:603–615. https://doi.org/10.1007/s10526-023-10232-3

[CIT0047] Patel K , Zhang Z-Q. 2017. Prey preference and reproduction of predatory mites, *Amblybromalus limonicus* and *Neoseiulus cucumeris*, on eggs and 1st instar nymphs of the tomato/potato psyllid. Int. J. Acarol. 43:468–474. https://doi.org/10.1080/01647954.2017.1349177

[CIT0048] R Core Team. 2024. R: a language and environment for statistical computing. 4.4.0. Vienna, Austria: R Foundation for Statistical Computing. https://www.R-project.org/

[CIT0049] Ramírez-Gil JG , López JH, Henao-Rojas JC. 2019. Causes of Hass avocado fruit rejection in preharvest, harvest, and packinghouse: economic losses and associated variables. Agronomy. 10:8. https://doi.org/10.3390/agronomy10010008

[CIT0050] Reis PR , Sousa EO, Teodoro AV, et al 2003. Effect of prey density on the functional and numerical responses of two species of predaceous mites (Acari: Phytoseiidae). Neotrop. Entomol. 32:461–467. https://doi.org/10.1590/s1519-566x2003000300013

[CIT0051] Reis PR , Teodoro AV, Pedro Neto M, et al 2007. Life history of *Amblyseius herbicolus* (Chant)(Acari: Phytoseiidae) on coffee plants. Neotrop. Entomol. 36:282–287. https://doi.org/10.1590/s1519-566x2007000200016 17607463

[CIT0052] Richardson J. 2020. Selectapref: analysis of field and laboratory foraging. R package 0.1.2. https://CRAN.R-project.org/package=selectapref

[CIT0053] Rodríguez-Cruz FA , Venzon M, Pinto CMF. 2013. Performance of *Amblyseius herbicolus* on broad mites and on castor bean and sunnhemp pollen. Exp. Appl. Acarol. 60:497–507. https://doi.org/10.1007/s10493-013-9665-y 23417701

[CIT0054] Sabelis M. 1990. How to analyse prey preference when prey density varies? A new method to discriminate between effects of gut fullness and prey type composition. Oecologia. 82:289–298. https://doi.org/10.1007/BF00317473 28312701

[CIT0055] Sabelis MW , Bakker FM. 1992. How predatory mites cope with the web of their tetranychid prey: a functional view on dorsal chaetotaxy in the Phytoseiidae. Exp. Appl. Acarol. 16:203–225. https://doi.org/10.1007/bf01193804

[CIT0056] Samaras K , Pappas ML, Fytas E, et al 2015. Pollen suitability for the development and reproduction of *Amblydromalus limonicus* (Acari: Phytoseiidae). BioControl. 60:773–782. https://doi.org/10.1007/s10526-015-9680-5

[CIT0057] Samaras K , Pappas ML, Fytas E, et al 2019. Pollen provisioning enhances the performance of *Amblydromalus limonicus* on an unsuitable prey. Front. Ecol. Evol. 7:122. https://doi.org/10.3389/fevo.2019.00122

[CIT0058] Schoeller EN , McKenzie CL, Osborne LS. 2020. Comparison of the phytoseiid mites *Amblyseius swirskii* and *Amblydromalus limonicus* for biological control of chilli thrips, *Scirtothrips dorsalis* (Thysanoptera: Thripidae). Exp. Appl. Acarol. 82:309–318. https://doi.org/10.1007/s10493-020-00556-5 33025240

[CIT0059] Shimoda T , Kishimoto H, Takabayashi J, et al 2010. Relationship between the ability to penetrate complex webs of Tetranychus spider mites and the ability of thread-cutting behavior in phytoseiid predatory mites. Biol. Control. 53:273–279. https://doi.org/10.1016/j.biocontrol.2010.02.007

[CIT0060] Steven D. 2004. Control of six-spotted mite, *Eotetranychus sexmaculatus*. *New Zealand Avocado Growers’ Association Annual Research Report Volume 4, 2004.* https://www.avocadosource.com/Journals/NZAGA/NZAGA_2004/NZAGA_2004_13.pdf

[CIT0061] Stevens P , Jamieson L, Cave J. 2001. Comparative toxicity of pesticides to the sixspotted mite *Eotetranychus sexmaculatus* (Riley) (Acari: Tetranychidae) on avocados. *New Zealand Avocado Growers’ Association Annual Research Report Volume 1, 2001.* http://209.143.153.251/Journals/NZAGA/NZAGA_2001/NZAGA_2001_02.pdf

[CIT0062] Talavera A , Gonzalez-Fernandez J, Carrasco-Pancorbo A, et al 2023. Avocado: agricultural importance and nutraceutical properties. In: Kole C editor. Compendium of crop genome designing for nutraceuticals. Springer. p. 1–19.

[CIT0063] Tixier M-S. 2018. Predatory mites (Acari: Phytoseiidae) in agro-ecosystems and conservation biological control: a review and explorative approach for forecasting plant-predatory mite interactions and mite dispersal. Front. Ecol. Evol. 6:192. https://doi.org/10.3389/fevo.2018.00192

[CIT0064] Tomkins A. 2002. Sustainable management of sixspotted spider mite (*Eotetranychus sexmaculatus* (Riley)) on avocados. *New Zealand Avocado Growers’ Association Annual Research Report Volume 2, 2002.* http://209.143.153.251/Journals/NZAGA/NZAGA_2002/NZAGA_2002_04.pdf

[CIT0065] Torres E , Álvarez-Acosta C, del Pino M, et al 2023. Economic impact of the Persea mite in Spanish avocado crops. Agronomy. 13:668. https://doi.org/10.3390/agronomy13030668

[CIT0067] Wang X , Xiang Y, Sun M, et al 2023. Transcriptomic and metabolomic analyses reveals keys genes and metabolic pathways in tea (*Camellia sinensis*) against six-spotted spider mite (*Eotetranychus Sexmaculatus*). BMC Plant Biol. 23:638. https://doi.org/10.1186/s12870-023-04651-8 38072959 PMC10712147

[CIT0066] Wang J , Zhang K, Li L, et al 2024. Development and reproduction of four predatory mites (Parasitiformes: Phytoseiidae) feeding on the spider mites *Tetranychus evansi* and *T. urticae* (Trombidiformes: Tetranychidae) and the dried fruit mite *Carpoglyphus lactis* (Sarcoptiformes: Carpoglyphidae). Syst. Appl. Acarol. 29:269–284. https://doi.org/10.11158/saa.29.2.7

[CIT0068] Wickham H. 2016. Ggplot2: elegant graphics for data analysis. R package. New York: Springer-Verlag. https://ggplot2.tidyverse.org

[CIT0069] Xiao Y , Fadamiro HY. 2010. Functional responses and prey-stage preferences of three species of predacious mites (Acari: Phytoseiidae) on citrus red mite, *Panonychus citri* (Acari: Tetranychidae). Biol. Control. 53:345–352. https://doi.org/10.1016/j.biocontrol.2010.03.001

[CIT0070] Xin T-R , Zhang Z-Q. 2021. Suitability of pollen as an alternative food source for different developmental stages of *Amblyseius herbicolus* (Chant) (Acari: Phytoseiidae) to facilitate predation on whitefly eggs. Acarologia. 61:790–801. https://doi.org/10.24349/biv1-2hen

[CIT0071] Zhang K , Zhang Z-Q. 2021. The dried fruit mite *Carpoglyphus lactis* (Acari: Carpoglyphidae) is a suitable alternative prey for *Amblyseius herbicolus* (Acari: Phytoseiidae). Syst. Appl. Acarol. 26:2167–2176. https://doi.org/10.11158/saa.26.11.15

[CIT0072] Zhang K , Zhang Z-Q. 2022. *Amblyseius herbicolus* mothers prefer to oviposit near eggs of non-kin in the absence of prey *Carpoglyphus lactis* (Acari: Phytoseiidae, Carpoglyphidae). Syst. Appl. Acarol 27:2347–2354. https://doi.org/10.11158/saa.27.11.16

